# 637. Treatment Outcomes in Secondary Analysis Populations of Adult Patients the ALLIUM Phase 3 study Comparing Cefepime-Enmetazobactam to Piperacillin-Tazobactam for Complicated Urinary Tract Infections (cUTI) or Acute Pyelonephritis (AP)

**DOI:** 10.1093/ofid/ofab466.834

**Published:** 2021-12-04

**Authors:** Keith S Kaye, Adam Belley, Philip Barth, Omar Lahlou, Patrick Velicitat

**Affiliations:** 1 University of Michigan Medical School, Ann Arbor, MI; 2 Allecra Therapeutics SAS, Beaconsfield, Quebec, Canada

## Abstract

**Background:**

Superior treatment outcomes were observed with the β-lactam/β-lactamase inhibitor combination of cefepime-enmetazobactam (FPE) compared to piperacillin-tazobactam (PTZ) in the primary efficacy population (m-MITT) of the ALLIUM phase 3 study of adult patients with cUTI/AP. We present here the outcomes in the microbiologically evaluable (ME) and ME+Resistant (ME+R) populations.

**Methods:**

1034 cUTI/AP patients randomized 1:1 in a double-blind, multicenter trial received either 2 g cefepime/0.5 g enmetazobactam or 4 g piperacillin/0.5 g tazobactam q8h by 2h infusion for 7 to 14 days. Patients in m-MITT had a Gram-negative urinary baseline pathogen (BP) at >10^5^ CFU/ml with FPE MIC ≤8 µg/ml and PTZ MIC ≤64 µg/ml. ME included patients in m-MITT who received ≥15 consecutive doses of study drug or were classified as clinical failures after receiving ≥9 doses; had a clinical assessment at test-of-cure (TOC) unless clinical failure occurred earlier; did not receive concomitant antibiotics with a non-study agent; and did not have any other protocol violation. ME+R included patients in ME along with those who had BP resistant to either FPE (MIC >8 µg/ml) or PTZ (MIC >64 µg/ml), or a missing MIC value. Overall success was the composite of clinical cure and microbiological eradication (< 10^3^ CFU/ml in urine). Two-sided 95% confidence interval (CI) were computed using the stratified Newcombe method.

**Results:**

In the ME population, superiority in overall success of FPE (87.0%; 268/308) compared to PTZ (65.4%; 195/298) was demonstrated as the lower bound of the CI (16.6%) of the treatment difference (TD; 23.3%) was greater than 0 (Table). Higher rates of microbiological eradication with FPE contributed to the superior treatment outcomes. In the ME+R population in which BP susceptibility was not an exclusion criterion, favorable outcomes with FPE in overall success (TD 21.6%; 95% CI [15.3, 27.8]) and microbiological eradication (TD 21.0%; 95% CI [14.8, 27.0]) were also observed.

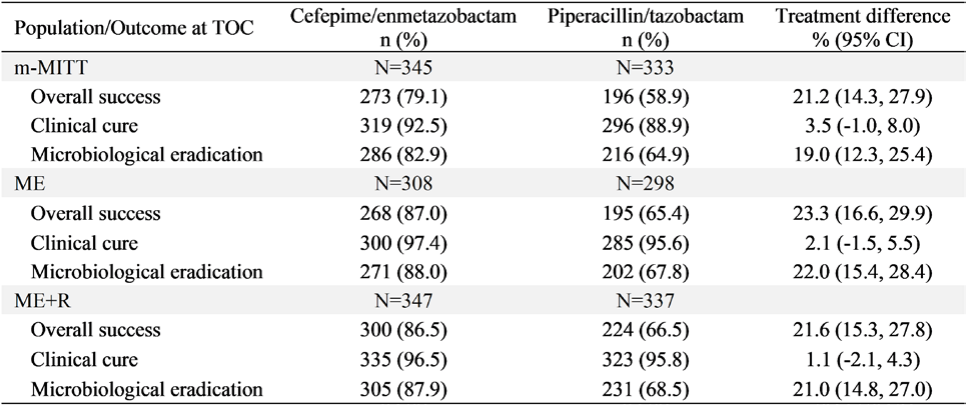

**Conclusion:**

The confirmation of superior treatment outcomes with FPE in the ME and ME+R populations supports the robustness of the corresponding superiority observed in adult cUTI/AP patients in m-MITT.

**Disclosures:**

**Adam Belley, PhD**, **Allecra Therapeutics SAS** (Consultant) **Philip Barth, MD**, **Allecra Therapeutics SAS** (Consultant) **Omar Lahlou, PhD**, **Allecra Therapeutics SAS** (Employee) **Patrick Velicitat, MD**, **Allecra Therapeutics SAS** (Employee)

